# A Longitudinal Study on Online Sexual Engagement, Victimization, and Psychosocial Well-Being

**DOI:** 10.3389/fpsyg.2021.674072

**Published:** 2021-12-08

**Authors:** Felix Reer, Ruth Wendt, Thorsten Quandt

**Affiliations:** ^1^Department of Communication, University of Münster, Münster, Germany; ^2^Social Media Lab, Leibniz-Institut für Wissensmedien, Tübingen, Germany

**Keywords:** sexting, online sexual victimization, sexy self-presentation, anxiety, loneliness, depression, psychosocial well-being, longitudinal

## Abstract

Several cross-sectional studies have shown that online sexual engagement (OSE) in the form of sexting or sexy self-presentation on social media is associated with an increased risk of experiencing negative consequences, such as online sexual victimization (OSV) or lower levels of psychosocial well-being. However, representative and longitudinal studies are scarce. The current study follows three research goals: (1) examining the prevalence of OSE and OSV among a random-quota sample of 1,019 German Internet users aged 14–64 years, (2) examining gender and age-related differences in OSE and OSV, and (3) examining the longitudinal relationships between OSE, OSV, and psychosocial well-being over a period of 1 year. Our results indicate that OSE and OSV are relatively widespread: 17.7% of the participants had already experienced OSV, 25.3% indicated that they had presented themselves online in a sexualized manner at least once in the past 2 months, and 22.7% showed a certain willingness to engage in sexting. We found higher rates among the younger participants. However, to a certain degree, older individuals were also affected. Male participants showed higher sexting willingness and more often presented themselves in a sexualized manner than females, whereas only small differences related to OSV were found. Concerning relationships with psychosocial well-being, our cross-sectional results showed that OSE, OSV, and mental problems are intercorrelated. Furthermore, we detected a significant long-term relationship between higher sexting willingness at time 1 and more victimization experienced 1 year later, whereas no significant longitudinal associations with lower levels of psychosocial well-being were identified.

## Introduction

The sharing and publication of personal sexual content and information have recently garnered wide scientific and public attention (e.g., Del Rey et al., 2019; Burić et al., 2020; Cornelius et al., 2020; Gassó et al., 2020, 2021a,b; van Ouytsel et al., 2020; Barroso et al., 2021; Wachs et al., 2021). Practices, such as *sexting* (e.g., Wolak and Finkelhor, 2011; Döring, 2014) or sexualized forms of self-presentation on social media platforms (*sexy self-presentation*; e.g., van Oosten and Vandenbosch, 2017; van Ouytsel et al., 2020), are increasingly popular, especially among adolescents and young adults. These sexualized online media usage behaviors carry risks of misuse, as private and intimate material may be misunderstood or forwarded without knowledge and permissions and to an unlimited audience (Heirman and Walrave, 2008).

*Sexting* can broadly be defined as “the sharing of personal, sexually suggestive text messages, or nude or nearly nude photographs or videos *via* electronic devices” (Mori et al., 2020, p. 1103). According to recent meta-analyses (Madigan et al., 2018; Mori et al., 2020), sexting is a quite common behavior among the younger generation. For example, Mori et al. (2020) meta-analyzed 50 studies and found that on average, 38.3% within the age group of 18–29 years have already send a sexting message, 41.5% have received a sexting message, and 47.7% have engaged in reciprocal sexting. Further, 15% were victims of non-consensual forwarding of sexting content (Mori et al., 2020).

*Sexy self-presentation* refers to sexualized forms of self-presentation on social media platforms, such as Instagram or Facebook, and, for example, “includes posting images in which someone is scarcely dressed, has a sexy gaze or in which sexual willingness is suggested” (van Ouytsel et al., 2020, p. 1/15). In comparison to sexting, which typically occurs in inter-personal conversations and is quite explicit, sexy self-presentation is a more suggestive and less private form of sexual self-expression (van Oosten and Vandenbosch, 2017). Similar to studies on sexting, research has found that engagement in sexy self-presentation is relatively widespread among adolescents (Festl et al., 2019; van Ouytsel et al., 2020). For example, Festl et al. (2019) conducted a survey among German Internet users aged 14–20 years and found that 35% of the participants had made experiences with sexy self-presentations within the last 2 months.

Both sexting and sexy self-presentation have been considered sexual risk behaviors that may result in severe negative consequences, such as cyberbullying, sexual victimization, or different forms of psychological distress (e.g., Klettke et al., 2014; Gámez-Guadix et al., 2015; Festl et al., 2019; Gassó et al., 2019, 2020, 2021b; Mori et al., 2019; Burić et al., 2020; Barroso et al., 2021; García-González et al., 2021; Lu et al., 2021; Tamarit et al., 2021; Wachs et al., 2021). Indeed, several empirical studies have identified direct relationships between engagement in sexting and decreased mental health (Klettke et al., 2014; Gassó et al., 2019; Mori et al., 2019). For example, Frankel et al. (2018) surveyed 6,021 high school students and found that consensual as well as non-consensual sexting is positively associated with suicide attempts, self-harm, and depression symptoms. The finding that sexting is positively related to higher levels of depression symptomatology has been confirmed by several studies (e.g., Temple et al., 2014; van Ouytsel et al., 2014; Chaudhary et al., 2017; Gámez-Guadix and de Santisteban, 2018; Festl et al., 2019; Kim et al., 2020). Sexting has also been found to be positively related to anxiety (Chaudhary et al., 2017), emotional problems (Ševčíková, 2016), low self-esteem (Ybarra and Mitchell, 2014), and increased conduct disorder scores (Kim et al., 2020).

A plausible explanation for these findings may be that engagement in sexting can increase the risk of unintended sexual attention, unpleased contacts, and, in general, being victimized online (Gámez-Guadix et al., 2015). Accordingly, sexting and sexualized forms of online self-presentation have been found to be positively associated with a higher risk of online sexual victimization (OSV) and cyberbullying (e.g., Reyns et al., 2013; Gámez-Guadix et al., 2015; Medrano et al., 2018; Festl et al., 2019). Some studies found that victimization experiences mediate the relationship between online sexual engagement (OSE) and psychological distress (Medrano et al., 2018; Festl et al., 2019). For example, Medrano et al. (2018) showed that individuals who more often engage in sexting have a higher risk of becoming a victim of cyberbullying, which, in turn, is associated with higher scores in measurements of depression and suicidal ideation.

Although the number of studies on OSE and its correlates has increased in recent years, the existing research still suffers from some limitations. One issue concerns the fact that most studies were cross-sectional and thus did not allow examining the direction of the relationships between the measured constructs (Gassó et al., 2019; Mori et al., 2019). As pointed out by Gassó et al. (2019, p. 11), “some studies explored the psychological variables as predictors of sexting […], while others measured them as consequences of the behavior […].” In principal, both directions of relationships would make sense. On the one hand, it can be argued that behaviors, such as sexting and sexy self-presentation, increase the risk of experiencing negative psychosocial consequences. On the other hand, it can be argued that individuals with preexisting mental problems more often engage in online sexual risk behaviors owing to insufficient coping skills and a stronger need to feel popular and desired (Gassó et al., 2019). More longitudinal studies are needed to shed light on how OSE, OSV, and psychosocial well-being are related to each other over time (Mori et al., 2019).

Another limitation concerns the participant samples most studies are based on. A large majority of studies examine OSE among teenagers and adolescents (Klettke et al., 2014; Gassó et al., 2019; Mori et al., 2019, 2020) probably because younger users are considered an at-risk group for negative consequences. However, some empirical evidence suggests that sexting not only is a youth phenomenon but also is engaged in by quite a high percentage of middle-aged and older adults (Gámez-Guadix et al., 2015). More representative studies covering broader segments of society would be useful to allow for a better assessment of the prevalence of OSE and OSV (Klettke et al., 2014).

### The Current Study

The first goal of the current study was to investigate the prevalence of OSE (sexy self-presentation and sexting) and OSV based on a more representative and more heterogeneous sample than that typically used in this field of research. In particular, we aimed to examine OSE and OSV among a random-quota sample of German Internet users aged 14–64 years. Our first research question reads as follows:

RQ1: How prevalent are OSE and OSV among German Internet users aged 14–64 years?

Research among adolescents found that a higher willingness to engage in sexting is associated with older age (Klettke et al., 2014; Madigan et al., 2018), whereas studies that covered a broader age range showed the opposite effect (a higher willingness among younger participants; e.g., Gámez-Guadix et al., 2015). Concerning gender, few studies showed that females are more willing to send sexts (e.g., Mitchell et al., 2012; Ybarra and Mitchell, 2014), few indicated higher sexing willingness among males (e.g., West et al., 2014; Festl et al., 2019), and some found no significant differences between the genders (e.g., Kim et al., 2020). In general, females are often considered to have a high risk of experiencing negative consequences of OSE (e.g., Bianchi et al., 2019; Burić et al., 2020). Against this background, we examined whether age and gender-related differences in OSV and OSE were identifiable in the current random-quota-based study:

RQ2: Are there gender and age-related differences in OSV and OSE?

As outlined above, several cross-sectional studies identified positive associations between OSE, OSV, and decline in psychosocial well-being. However, the directions of these relationships often remained unclear because longitudinal studies are still scarce. Following a cross-lagged panel approach, the main goal of the current study therefore was to examine the longitudinal relationships between OSE, OSV, and psychosocial well-being. Based on existing cross-sectional research (Festl et al., 2019), we selected three different relevant indicators of psychosocial well-being, namely, depression/anxiety, life satisfaction, and loneliness.

RQ3: How are OSE, OSV, and psychosocial well-being related over time?

## Materials and Methods

### Participants and Procedures

The study was conducted as an online survey. We cooperated with a professional German survey research institute (adhering to the ICC/ESOMAR ethics code) that provided access to an online panel. A random-quota procedure with the criteria age, gender, living region, and education was applied to increase the representativeness of the sample. The initial sample (T1) was collected in 2018 and (after data cleansing) comprised of 1,019 German Internet users aged 14–64 years. The mean age of the initial sample was 41.30 years (*SD* = 13.91), and gender was almost equally distributed (50.9% males; 49.1% females).^1^

The second wave of the study was conducted 1 year later in 2019 (T2). In total, 586 usable datasets (58% of T1) were collected from the individuals who participated in both study waves.^2^

### Measurements

The different constructs were measured with translated versions of established instruments. German language versions of the scales measuring OSV, sexting willingness, and sexy self-presentation were adopted from Festl et al. (2019). To assess reliability, we calculated Cronbach’s alpha three times for each scale: for the initial sample of 1,019 German Internet users collected in 2018 (T1_initial_) and separately for those participants who took part in both study waves (T1_panel_ and T2_panel_).

#### Sexting Willingness

Participants’ willingness to engage in sexting was measured with items originally developed by van Oosten and Vandenbosch (2017). The participants indicated the likelihood that “they would send a picture *via* the Internet or text message of them being naked or almost naked, if this was asked of them by (a) their partner, (b) someone they are dating, (c) a friend, (d) a stranger, or (e) their ex-partner.” A seven-point scale ranging from 1 = “very unlikely” to 7 = “very likely” was used to rate the items (T1_initial_: *α* = 0.903; T1_panel_: *α* = 0.914; and T2_panel_: *α* = 0.909).

#### Sexy Self-Presentation

To get an impression of the participants’ self-presentation behaviors on social media, we adopted the sexy self-presentation scale by van Oosten and Vandenbosch (2017). The participants were asked “how often in the past 2 months they had uploaded pictures on their social media profile portraying themselves (a) with a sexy gaze, (b) with a sexy appearance, (c) scantily dressed (e.g., bathing suit or underwear), and (d) in a sexy posture.” Each of the items was answered on a scale ranging from 1 = “never” to 7 = “very often” (T1_initial_: *α* = 0.961; T1_panel_: *α* = 0.971; and T2_panel_: *α* = 0.960).

#### Online Sexual Victimization

We used the OSV scale developed by Gámez-Guadix et al. (2015) to measure how often the participants had negative sexuality-related experiences when using the Internet. The scale consisted of 10 items (e.g., “Somebody has disseminated or uploaded to the Internet photos or videos of you with erotic or sexual content without your consent”) that address three different dimensions of OSV experiences: threats and coercion, insistence, and the dissemination of personal sexual content/information without consent. A five-point scale was used to assess how often the participants had made such experiences online (0 = “never,” 1 = “1 or 2 times,” 2 = “3 or 4 times,” 3 = “5 or 6 times,” and 4 = “7 or more times”; T1_initial_: *α* = 0.969; T1_panel_: *α* = 0.972; and T2_panel_: *α* = 0.970).

#### Loneliness

Loneliness was measured with a German translation (Reer et al., 2019) of the short version (Hughes et al., 2004) of the revised UCLA loneliness scale (Russell et al., 1980). The scale consisted of three items (e.g., “How often do you feel that you lack companionship?”) that had to be rated from 1 = “never” to 4 = “often” (T1_initial_: *α* = 0.828; T1_panel_: *α* = 0.847; and T2_panel_: *α* = 0.850).

#### Life Satisfaction

The German version (Schumacher, 2003; Glaesmer et al., 2011) of the life satisfaction scale by Diener et al. (1985) was filled out by the participants using a seven-point scale ranging from 1 = “strongly disagree” to 7 = “strongly agree.” An example item of the scale reads as follows: “In most ways, my life is close to my ideal” (T1_initial_: *α* = 0.897; T1_panel_: *α* = 0.904; and T2_panel_: *α* = 0.932).

#### Depression/Anxiety

We measured the participants’ mental health using the patient health questionnaire (PHQ-4) by Kroenke et al. (2009) (German version by Löwe et al., 2010). The PHQ-4 asks to indicate how often someone experienced depression symptoms (e.g., “Feeling down, depressed, or hopeless”) and anxiety symptoms (e.g., “Feeling nervous, anxious, or on edge”) during the last 2 weeks. The four items (two per sub-dimension) were rated on a scale ranging from 0 = “not at all” to 3 = “nearly every day” and were composed to build a depression/anxiety index for each participant (T1_initial_: *α* = 0.891; T1_panel_: *α* = 0.903; and T2_panel_: *α* = 0.899).

#### Control Variables

Like previous studies (e.g., Festl et al., 2019), we considered control variables that might be relevant in the context of OSE and OSV. In addition to age and gender, we asked the participants to indicate their amount of Internet use (minutes) in a typical week (T1_initial_: *M* = 1,250.69, *SD* = 1,028.83; T1_panel_: *M* = 1,239.65, *SD* = 1,007.62). Further, we asked the participants about their sexual orientation using the following categories: “heterosexual,” “bisexual,” “gay/lesbian,” or “other” (following Savin-Williams, 2014). Following the approach by Festl et al. (2019), we dichotomized the responses to distinguish between heterosexual (=0; T1_initial_: 91.6%; T1_panel_: 92.0%) and non-heterosexual (=1; T1_initial_: 8.4%; T1_panel_: 8.0%) individuals.

### Data Analysis

Descriptive statistics concerning the prevalence of OSE and OSV (RQ1) were calculated based on the initial sample (T1_initial_). Following the approach of Festl et al. (2019), the reported percentages refer to the participants who at least once had made experiences with sexualized forms of self-presentation or OSV. For sexting, the percentage refers to those who showed willingness to engage in sexting by answering at least one item of the corresponding measurement as 5 or higher on the provided seven-point scale.

RQ2 asks about gender differences in OSE and OSV. We again followed the approach of Festl et al. (2019) and performed chi-square tests (prevalence rates) and independent *t*-tests (mean differences) to examine how OSE and OSV differed between gender and age groups. For all *t*-tests, bootstrapped CIs of the mean differences (2,000 samples, 95% bias-corrected and accelerated method) were additionally calculated to account for the non-normality of the data (Field, 2018). Cohen’s *d* was reported as a measure of the effect size.

Based on the panel data (T1_panel_ and T2_panel_), we used a cross-lagged model to examine the long-term relationships between OSV,^3^ sexting willingness, sexy self-presentation, and the three indicators of psychosocial well-being (RQ3). The model was estimated using the software R (FIML imputation) and the lavaan package (Rosseel, 2012). We used the MLR estimator, which features “maximum likelihood estimation with robust (Huber-White) standard errors and a scaled test statistic that is (asymptotically) equal to the Yuan-Bentler test statistic” (Rosseel, 2012, p. 27). Established criteria were applied to evaluate the fit of the model: TLI and CFI close to 0.95, RMSEA close to 0.06, and SRMR below 0.08 (Hu and Bentler, 1999).^4^

## Results

### Prevalence Rates

Concerning RQ1, a summary of our descriptive findings on the prevalence of OSE and OSV at T1 (*N* = 1,019) is provided in Table 1.

**Table 1:** Gender and age group differences in means and prevalence rates of sexting willingness, sexy self-presentation, and OSV.

	All complete datasets			Males			Females			Age: 14–35			Age: 36–64		
*N*	*M* (*SD*)	% (*n*)	*N*	*M* (*SD*)	% (*n*)	*N*	*M* (*SD*)	% (*n*)	*N*	*M* (*SD*)	% (*n*)	*N*	*M* (*SD*)	% (*n*)
Sexting Willingness (1–7)	987	1.72 (1.28)	22.7 (224)	499	2.00^*^ (1.46)	30.3^*^ (151)	488	1.42^*^ (0.99)	15.0^*^ (73)	363	2.06^*^ (1.46)	33.6^*^ (122)	624	1.51^*^ (1.12)	16.3^*^ (102)
Sexy Self-Presentation (1–7)	996	1.58 (1.26)	25.3 (252)	510	1.77^*^ (1.47)	28.8^*^ (147)	486	1.38^*^ (0.95)	21.6^*^ (105)	363	1.81^*^ (1.43)	34.7^*^ (126)	633	1.45^*^ (1.13)	19.9^*^ (126)
OSV (0–4)	974	0.17 (0.55)	17.7 (172)	493	0.22^*^ (0.67)	17.0 (84)	481	0.11^*^ (0.38)	18.3 (88)	356	0.27^*^ (0.67)	29.2^*^ 104	618	0.11^*^ (0.45)	11.0^*^ (68)

OSV: online sexual victimization. Results were calculated based on the initial sample at T1 (*N* = 1,019). Incomplete datasets were excluded. The reported prevalence rates refer to participants that at least once had made experiences with sexualized forms of self-presentation or OSV. For sexting, the percentage refers to those that showed a certain willingness to engage in sexting by answering at least one item of the corresponding measurement with 5 or higher on the provided seven-point scale.

* group differences are significant with *p* < 0.01.

According to our data, several participants already had experienced OSV (17.7%). Further, 25.3% indicated that they had presented themselves in a sexualized manner on social media at least once in the past 2 months, and 22.7% showed willingness (“rather probable,” “probable,” or “highly probable”) to engage in sexting.

RQ2 addressed age and gender differences in OSE and OSV.

We found that males scored higher on the instruments measuring sexting willingness (mean difference: 0.584 [BCa 95% CI:0.437, 0.735], *t* = 7.37, df = 879.01, *p* < 0.001, *d* = 0.467), engagement in sexy self-presentation (mean difference: 0.400 [BCa 95% CI:0.249, 0.559], *t* = 5.12, df = 875.93, *p* < 0.001, *d* = 0.321), and OSV (mean difference: 0.112 [BCa 95% CI:0.041, 0.183], *t* = 3.23, df = 788.01, *p* < 0.01, *d* = 0.205). Moreover, a higher percentage of males than females had presented themselves in a sexualized manner on social media (males: 28.8%; females: 21.6%; *χ*^2^ = 6.86, df = 1, *p* < 0.01) and showed a certain willingness to engage in sexting (males: 30.3%; females: 15.0%; *χ*^2^ = 32.93, df = 1, *p* < 0.001). No significant gender difference was found concerning the percentage of those who at least once had made a victimization experience (males: 17.0%; females: 18.3%; *χ*^2^ = 0.26, df = 1, *p* = 0.607).

Two age groups (group1: 14–35 years; group2: 36–64 years) were built to examine age differences. As illustrated in Table 1, we found that younger participants received higher scores on the scales measuring sexting willingness (mean difference: 0.545 [BCa 95% CI:0.357, 0.743], *t* = 6.15, df = 612.16, *p* < 0.001, *d* = 0.434), sexy self-presentation (mean difference: 0.362 [BCa 95% CI:0.203, 0.530], *t* = 4.15, df = 624.30, *p* < 0.001, *d* = 0.291), and OSV (mean difference: 0.165 [BCa 95% CI:0.094, 0.236], *t* = 4.14, df = 545.45, *p* < 0.001, *d* = 0.173). Further, a higher percentage of the younger participants showed a certain willingness to engage in sexting (group1: 33.6%; group2: 16.3%; *χ*^2^ = 38.98, df = 1, *p* < 0.001), and had made at least one experience with sexy self-presentation (group1: 34.7%; group2: 19.9%; *χ*^2^ = 26.76, df = 1, *p* < 0.001) and OSV (group1: 29.2%; group2: 11.0%; *χ*^2^ = 51.51, df = 1, *p* < 0.001).

### Cross-Lagged Panel Analysis

**Figure 1:**
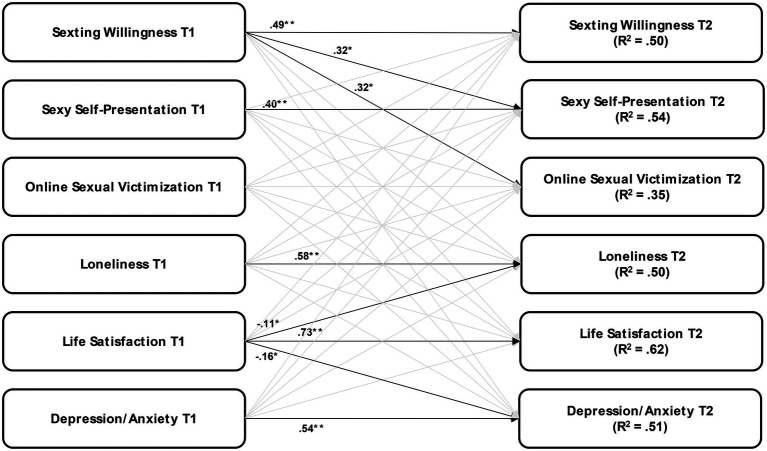
Cross-lagged panel model to explain the longitudinal relationships between online sexual engagement (OSE), online sexual victimization (OSV), and psychosocial well-being. *χ2(df)* = 2,062(1,070), *p* < 0.001, *CFI* = 0.951, *TLI* = 0.943, *RMSEA* = 0.047, and *SRMR* = 0.038. Indicated are the significant standardized path coefficients (*β*). ^**^p < 0.01 and ^*^p < 0.05. Non-significant paths are greyed out. Co-variances were calculated, but omitted for better overview. The model was additionally controlled for a person’s age, gender, sexual orientation, and amount of online use.

RQ3 was examined based on the subsample of participants who participated in both study waves (*N* = 586). A visualization of the cross-lagged analysis is shown in Figure 1. An overview of all the longitudinal relationships is provided in Table 2. In addition, the correlations between all latent and observed variables measured at T1_panel_ that were calculated as part of the cross-lagged model are reported in Table 3.

**Table 2:** Longitudinal relationships between online sexual engagement, victimization, and psychosocial well-being.

	Online Sexual Engagement and Victimization (T2_panel_)									Psychosocial Well-Being (T2_panel_)								
	Sexting Willingness			Sexy Self-Presentation			OSV			Depression/Anxiety			Loneliness			Life Satisfaction		
	*β*	*p*	*B (SE)*	*β*	*p*	*B (SE)*	*β*	*p*	*B (SE)*	*β*	*p*	*B (SE)*	*β*	*p*	*B (SE)*	*β*	*p*	*B (SE)*
**Control Variables (T1_panel_)**																		
Age	**−0.09**	**0.006**	**−0.10 (0.04)**	−0.04	0.204	−0.05 (0.04)	**−0.08**	**0.006**	**−0.05 (0.02)**	**−0.07**	**0.029**	**−0.05 (0.02)**	**−0.11**	**0.003**	**−0.05 (0.02)**	0.03	0.289	0.05 (0.04)
Male Gender	**0.07**	**0.045**	**0.16 (0.08)**	0.04	0.222	0.09 (0.08)	−0.00	0.928	−0.00 (0.04)	**−0.07**	**0.041**	**−0.10 (0.05)**	−0.05	0.135	−0.05 (0.03)	0.00	0.922	0.01 (0.09)
Non-Heterosexual	0.06	0.129	0.26 (0.17)	0.03	0.408	0.14 (0.17)	0.00	0.935	0.01 (0.08)	0.02	0.618	0.06 (0.12)	0.04	0.293	0.08 (0.07)	−0.03	0.381	−0.18 (0.20)
Internet Minutes	0.07	0.127	0.08 (0.05)	0.04	0.322	0.05 (0.05)	0.01	0.856	0.01 (0.03)	0.01	0.782	0.01 (0.03)	−0.04	0.392	−0.02 (0.02)	−0.04	0.382	−0.06 (0.07)
**Online Sexual Engagement and Victimization (T1_panel_)**																		
Sexting Willingness	**0.49**	**0.003**	**0.46 (0.15)**	**0.32**	**0.017**	**0.31 (0.13)**	**0.32**	**0.046**	**0.15 (0.08)**	0.05	0.644	0.03 (0.06)	0.08	0.410	0.03 (0.04)	0.02	0.825	0.03 (0.12)
Sexy Self-Presentation	0.20	0.162	0.17 (0.13)	**0.40**	**0.001**	**0.36 (0.11)**	0.10	0.464	0.04 (0.06)	−0.01	0.938	−0.00 (0.05)	−0.02	0.864	−0.01 (0.04)	−0.03	0.708	−0.04 (0.10)
OSV	−0.01	0.898	−0.03 (0.19)	0.02	0.825	0.04 (0.20)	0.20	0.156	0.19 (0.13)	−0.02	0.750	−0.03 (0.09)	0.05	0.478	0.04 (0.06)	−0.02	0.702	−0.06 (0.15)
**Psychosocial Well-Being (T1_panel_)**																		
Depression/Anxiety	−0.05	0.496	−0.07 (0.11)	−0.04	0.560	−0.06 (0.10)	−0.02	0.749	−0.02 (0.06)	**0.54**	**0.000**	**0.53 (0.07)**	0.03	0.685	0.02 (0.05)	−0.10	0.125	−0.20 (0.13)
Loneliness	0.05	0.313	0.11 (0.10)	0.06	0.176	0.13 (0.10)	0.02	0.674	0.02 (0.05)	0.10	0.060	0.13 (0.07)	**0.58**	**0.000**	**0.55 (0.05)**	−0.01	0.863	−0.02 (0.13)
Life Satisfaction	−0.09	0.119	−0.07 (0.05)	−0.03	0.601	−0.02 (0.04)	−0.09	0.089	−0.04 (0.02)	**−0.16**	**0.003**	**−0.08 (0.03)**	**−0.11**	**0.041**	**−0.04 (0.02)**	**0.73**	**0.000**	**0.78 (0.06)**
*R* ^2^	0.50			0.54			0.35			0.51			0.50			0.62		

*N* = 586. OSV: online sexual victimization Age and Internet minutes were centered. Significant effects are marked bold (*p* < 0.05).

**Table 3:** Cross-sectional correlations between latent and observed variables in the cross-lagged model.

	1	2	3	4	5	6	7	8	9	10
Age	-									
Male	0.006	-								
Non-Heterosexual	−0.053	0.061	-							
Internet Minutes	−0.013	0.107^**^	0.118	-						
Sexting Willingness	−0.235^**^	0.164^**^	0.077	−0.036	-					
Sexy Self-Presentation	−0.164^**^	0.157^**^	0.126^*^	0.020	0.846^**^	-				
OSV	−0.197^**^	0.131^**^	0.056	−0.000	0.713^**^	0.674^**^	-			
Depression/Anxiety	−0.177^**^	0.002	0.070	0.056	0.468^**^	0.473^**^	0.457^**^	-		
Loneliness	−0.128^**^	0.064	−0.014	0.045	0.417^**^	0.391^**^	0.332^**^	0.584^**^	-	
Life Satisfaction	−0.060	−0.008	0.035	−0.133^**^	0.112^**^	0.141^**^	0.057	−0.447^**^	−0.361^**^	-

T1_panel_ correlations calculated as part of the cross-lagged model. *N* = 586. OSV: online sexual victimization.

** *p* < 0.01.

* *p* < 0.05.

The analysis revealed several significant correlations between the main study variables at T1_panel_ (see Table 3). Both increased willingness to engage in sexting and more frequent use of social media for sexualized self-presentation were positively correlated with an increased risk of being victimized online. Further, sexy self-presentation and sexting willingness were both positively related to more loneliness and more depression/anxiety and showed small positive relationships with life satisfaction. Regarding experiences of OSV, we found significant positive correlations with higher levels of loneliness and depression/anxiety. No significant cross-sectional relationship was found between OSV and general life satisfaction.

Despite these cross-sectional findings, we found that the longitudinal relationships between the main study variables were mostly non-significant (see Figure 1; Table 2). Psychosocial well-being at T1 did not predict OSE or OSV at T2; and OSE and OSV at T1 did not predict psychosocial well-being at T2. We, however, found that more sexting willingness at T1 was related to more victimization experiences at T2. Further, significant longitudinal relationships were identified for younger age (higher sexting willingness and more OSV at T2) and male gender (more sexting willingness at T2). In the model, the autoregressive path from OSV at T1 to OSV at T2 was non-significant. An examination of the bivariate relationship showed a moderate positive relationship (*β* = 0.49, *p* < 0.001).

## Discussion

Although many existing studies have investigated OSE and OSV among (convenience) samples of adolescents and younger adults (Klettke et al., 2014; Madigan et al., 2018; Mori et al., 2020), the prevalence of OSE and OSV among older segments of society has so far remained understudied. Thus, the first aim of the current study was to examine the prevalence of OSE and OSV among a more heterogeneous sample than that typically used in this field of research. The initial random-quota sample of the current study comprised 1,019 German Internet users aged 14–64 years. Our data showed that almost 20% of the participants had made at least one experience with OSV, more than 20% showed a certain willingness to engage in sexting, and more than 25% had presented themselves in a sexualized manner on social media in the past 2 months.

The second aim of the current study was to examine gender and age differences in OSE and OSV. Research among adolescents showed that sexting willingness increases with age (Klettke et al., 2014; Madigan et al., 2018). However, it was considered that this effect might disappear or even change its direction within samples covering a broader age range (Klettke et al., 2014). Affirming this assumption and previous findings (Gámez-Guadix et al., 2015), our results showed that younger participants aged 14–35 years had higher OSE and had encountered more OSV than participants aged 36–64 years. However, our data also confirmed that behaviors, such as sexting and sexy self-presentation, as well as experiences with OSV, to a lower extend also occurred among middle-aged and older adults. Thus, future research should give more attention to these older age groups.

Concerning gender differences, we found that males were more willing to engage in sexting and more often had presented themselves in a sexualized manner on social media than females. Females have often been discussed to have a higher risk of experiencing negative consequences of sexting, such as cyberbullying, sexual harassment, insults, or online shaming (Ringrose et al., 2013; Lippman and Campbell, 2014; West et al., 2014; Bianchi et al., 2019; Burić et al., 2020). This might be a reason why some recent studies solely focused on girls (e.g., Bianchi et al., 2019; Burić et al., 2020). According to our data, males showed slightly higher mean scores on the scale measuring the frequency of OSV experiences, whereas no significant gender difference was found when comparing the percentage of individuals who had made at least one experience with OSV. Thus, our results indicate that OSV is not a topic that only concerns females. Research on all sexes is necessary to understand the issue in all its breadth.

The main aim of the current study was to examine the relationship between OSE, OSV, and psychosocial well-being. Several existing cross-sectional studies have shown that OSE is related to decline in mental health and well-being (Klettke et al., 2014; Gassó et al., 2019; Mori et al., 2019). Confirming these findings, we found that sexting willingness and sexy self-presentation were both positively related to higher levels of depression/anxiety and loneliness. Further, we found that both examined forms of OSE showed positive cross-sectional associations with a higher risk of being victimized online, which is also in line with several previous studies (e.g., Reyns et al., 2013; Gámez-Guadix et al., 2015; Medrano et al., 2018; Festl et al., 2019). Further, OSV was positively related to more loneliness and more depression/anxiety. However, OSV does not seem to threaten general life satisfaction because no significant relationship between these constructs was identified. In fact, life satisfaction showed small significant positive relationships with OSE, which might indicate that individuals more satisfied with their lives act more confident online.

Concerning the long-term relationship between sexting and psychosocial well-being, the results of the few existing studies are rather heterogeneous. For example, Chaudhary et al. (2017) surveyed a sample of middle school students from Texas and found that engagement in sexting predicted higher levels of depression and anxiety experienced 1 year later. In a similar study among Spanish adolescents, Gámez-Guadix and De Santisteban (2018) found that higher levels of depression at baseline predicted more sexting 1 year later, whereas self-esteem was no significant predictor of later OSE. In a recent study among adolescent girls covering a period of 20 months, Burić et al. (2020) found that sexting and psychological well-being were unrelated over time.

We found that neither sexting willingness nor sexy self-presentation showed any significant longitudinal relationship with depression/anxiety, loneliness, and life satisfaction. This can be interpreted as a hint that OSE does not threaten mental health in the long run and that lower levels of psychosocial well-being do not necessarily increase the likelihood for later OSE. The discrepancies with some existing studies may be attributable to the different sampling strategies (convenience adolescent samples vs. random-quota representative sample), different analysis strategies, or different measurements used. In particular, it might be possible that longitudinal relationships between OSE and psychosocial well-being primarily occur among particular groups of individuals (e.g., very young adolescents), which should be examined in more detail in future studies.

Few existing longitudinal studies among adolescents found reciprocal relationships between sexting and victimization experiences – that is, sexting was found to increase the risk to later being a victim of unwanted sexual solicitation and cyberbullying, and cyberbullying and sexual solicitation victimization were identified as significant predictors of later engagement in sexting (Gámez-Guadix and Mateos-Pérez, 2019; van Ouytsel et al., 2019). In the current study, we found that sexting willingness at T1 was a significant predictor of OSV measured 1 year later, whereas the reverse path (OSV at T1 to later sexting willingness) was non-significant. Concerning sexy self-presentation, no significant longitudinal relationships with OSV were found. These findings indicate that sexting is indeed a longitudinal risk factor for victimization, also among a random-quota sample spanning a wider age range than that typically covered in existing works. In contrast, sexualized forms of social media self-presentation seem to be less risky from a long-term perspective, which might be explained by the fact that this form of OSE is less intimate and explicit than sexting (van Oosten and Vandenbosch, 2017). Notably, higher levels of OSV showed no long-term relationship with lower psychosocial well-being.

Research on cyberbullying suggests that victimization experiences are only moderately stable over time (e.g., Marciano et al., 2020), which might be explained by specific characteristics of online media (such as the high anonymity). We found that (despite a significant bivariate relationship) OSV measured at T1 did not significantly predict OSV measured at T2 in the cross-lagged model. A plausible explanation might be that the perception of what was enforced behavior and what was done out of free will may change over time. Further, it might be difficult for victims to remember the exact number of such incidents. An interesting yet challenging task for future studies would be to examine what role psychological defense mechanisms, such as repression and denial play, in the context of OSV and how this could influence study results.

Our study is subject to some limitations. As is the case with every survey study, we cannot completely rule out that social desirability influenced response behaviors. Therefore, studies based on behavioral data would be desirable. Further, the dropout in participants between study waves may influence the representativeness of the longitudinal data. The final panel sample was not sufficiently large to allow for conducting more complex analyses (e.g., multi-group longitudinal analyses between genders or sexual orientations).

## Conclusion

Taken together, the current random-quota-based study showed that sexting, sexy self-presentation, and OSV are relatively widespread among German Internet users. According to our data, OSE and victimization experiences concern both males and females and, to a certain degree, also occur among older individuals. There have been lively debates among scholars about whether behaviors, such as sexting, are common sexual expressions in a digitalized world or whether they are deviant practices that expose individuals to unpredictable risks with consequences for their mental health and well-being (Burić et al., 2020). The results of our study paint a rather heterogeneous picture. Our cross-sectional results confirm previous findings that OSE, OSV, loneliness, and mental problems are intercorrelated (e.g., Festl et al., 2019; for overviews, see Klettke et al., 2014; Gassó et al., 2019; Mori et al., 2019). Concerning long-term associations, we detected a significant relationship between sexting willingness at T1 and more victimization experienced 1 year later, whereas no significant longitudinal associations with lower levels of psychosocial well-being were identified. However, note that the findings might be very different for specific and more vulnerable groups, such as children, young adolescents, or persons with specific sexual orientations.

## Data Availability Statement

The datasets presented in this article are not readily available because they are part of a larger representative survey study that covers several different topics and is subject to further analysis in other contexts. However, the raw data supporting the conclusions of this article are available to qualified researchers, upon reasonable request. Requests to access the datasets should be directed to FR, felix.reer@uni-muenster.de.

## Ethics Statement

Ethical review and approval was not required for the study on human participants in accordance with the local legislation and institutional requirements. The study was conducted in cooperation with a professional survey research institute adhering to the ICC/ESOMAR ethics code for social research and data analytics. All participants were informed, agreed to participate in the study and had the right to opt-out at any time. All data was collected in anonymized form. The authors had no access to any identifying information. Written informed consent from the participants’ legal guardian/next of kin was not required to participate in this study in accordance with the national legislations in Germany and the institutional requirements.

## Author Contributions

RW and FR conceptualized the study. FR administrated the project, coordinated the cooperation with the survey research institute, wrote the manuscript, and conducted parts of the statistical analyses. RW conducted parts of the statistical analyses and contributed to the writing of the manuscript. TQ obtained the funding and supported the conceptualization, analysis, and writing process. All authors contributed to the article and approved the submitted version.

## Funding

Parts of the research leading to these results have received funding from the Daimler and Benz Foundation *via* the project “Internet und seelische Gesundheit” (“Internet and Mental Health”). The sponsor had no role in the study design, the collection, analysis, interpretation of data, the writing of the report, or the decision to submit the article for publication.

## Conflict of Interest

The authors declare that the research was conducted in the absence of any commercial or financial relationships that could be construed as a potential conflict of interest.

## Publisher’s Note

All claims expressed in this article are solely those of the authors and do not necessarily represent those of their affiliated organizations, or those of the publisher, the editors and the reviewers. Any product that may be evaluated in this article, or claim that may be made by its manufacturer, is not guaranteed or endorsed by the publisher.

## Footnotes

^1^Comparing the sample characteristics with statistics on the German general population (Destatis – Statistisches Bundesamt, 2021) shows quite a close match cornering gender (population-wide ratio in 2019: 49.3% males and 50.6% females) and age (population-wide mean age in 2019: 44.5 years).

^2^Comparing the panel respondents (*n* = 586) with those who only participated in the first wave (*n* = 433), we found that the panel respondents were older (*M* = 43.75 compared to *M* = 37.99 years; mean difference: −5.76 [BCa 95% CI:-7.50, −4.01], *t* = −6.47, df = 816.31, *p* < 0.01, *d* = −0.423) and that the panel contained more male than female respondents (54.4% compared to 46.2%; *χ*^2^ = 6.78, df = 1, *p* < 0.01). There were no significant differences regarding the amount of respondents Internet use or their sexual orientation.

^3^Note that item parceling was used for the construct OSV.

^4^To analyze the effects over time, it is necessary to measure identical constructs at both panel waves. For each reflective construct, we conducted measurement invariance analyses prior to the main data analyses using the *longInvariance* function of the *semTools* package and checked whether removing the equality constraints on factor loadings would significantly improve the fit indices for each model: comparative fit index (CFI, increase of ≥0.010) and root mean square error of approximation (RMSEA, decrease of ≥0.015; see Chen, 2007). Measurement invariance could be confirmed for all constructs.
